# One year experience of *Achromobacter* bacteremia at a tertiary care hospital in Northern India

**DOI:** 10.1099/acmi.0.000588.v3

**Published:** 2023-09-13

**Authors:** Mitra Kar, Romya Singh, Nidhi Tejan, Ashima Jamwal, Akanksha Dubey, Radhika Chaudhary, Chinmoy Sahu, Sangram Singh Patel, Pooja Kumari, Malay Ghar

**Affiliations:** ^1^​ Department of Microbiology, Sanjay Gandhi Postgraduate Institute of Medical Sciences, Lucknow, Uttar Pradesh-226014, India

**Keywords:** Gram-negative, non-fermentative bacteria, *Achromobacter*, catheters, *Achromobacter *bacteremia, antibiotic therapy

## Abstract

**Introduction.:**

*
Achromobacter* is a Gram-negative, motile, obligate aerobic and non-fermentative bacterium. It is an emerging pathogen in the hospital environment as it is frequently found in various solutions.

**Hypothesis/Gap Statement.:**

Information about the incidence and risk factors of *Achromobacter* bacteremia from India is limited.

**Aim.:**

We conducted this study to identify the risk factors and underlying conditions predisposing to bacteremia by *Achromobacter* spp. and analyse the antibiotic resistance pattern of the isolates.

**Methodology.:**

We performed a retrospective observational study where automated blood cultures positive for *Achromobacter* spp. were assessed for clinical characteristics and antibiotic susceptibility patterns from January 2022 to December 2022 in the microbiology laboratory of a tertiary care centre in Northern India.

**Results.:**

A total of 14 cases (14/2435, 0.57 %) of *Achromobacter* spp. were identified from bloodstream infections in one year. The mean age of the patients was 37.59±23.17 years with a male predominance (8/14, 57.1 %). All patients were managed on intravenous antibiotics and intravenous access as peripheral line catheters and only 5(5/14, 35.7 %) patients were managed on central line catheters. The isolates were found highly susceptible to ticarcillin-clavulanic acid (14/14, 100.0 %) followed by fluoroquinolones (12/14, 85.72 %) and trimethoprim-sulphamethoxazole (12/14, 85.72 %). Only 57.14 % (8/14, 57.14 %) of the patients were susceptible to piperacillin-tazobactam. The all-cause 40 day mortality was observed in 35.7 % (5/14, 35.7 %) with two deaths that were directly attributable to sepsis.

**Conclusion.:**

This study provides insight into the incidence of *Achromobacter* bacteremia at our centre and the necessary antibiotic therapy to combat it.

## Data Summary

No supporting data in publicly available repositories (where available and appropriate) has been added to the main manuscript.

## Introduction

*Achromobacter* is a Gram-negative, motile, obligate aerobic and non-fermentative bacteria. It is catalase and oxidase positive, citrate positive, and indole and urease negative [1]. *Achromobacter species* has various subspecies, among which the two most important clinically isolated species are *xylosoxidans* and *denitrificans* [2]. *Achromobacter xylosoxidans* was first described by Yabuuchi and Ohyama *et al*. from an ear pus sample obtained from a patient suffering from chronic ear infections [3].

*Achromobacter* spp. is often misidentified by other non-lactose fermenting, Gram-negative bacteria, mostly *Pseudomonas* spp., and with rare pathogens (i.e. *Pandoraea* spp. and *Ralstonia* spp.) with the use of conventional methods due to similar biochemical reactions [4–6]. *Achromobacter* is a multidrug-resistant (MDR) opportunistic pathogen [7]. It is known to be an emerging pathogen in the hospital environment as it is frequently found as a contaminant in various solutions used in hospitals including water from nebulizers, dialysis water, incubators, extracorporeal circulation system and also in poorly preserved antiseptic and disinfectant solutions due to resistance to these agents [8, 9]. *Achromobacter* spp. most predominantly *Achromobacter xylosoxidans *, is recovered as a chronic respiratory pathogen from patients with cystic fibrosis (CF) attributable to poor patient outcome [10–12]. Many authors have reported a high prevalence of *Achromobacter* in hospital settings as a result of cross-contamination [12, 13]. *Achromobacter* spp. is an MDR pathogen with intrinsic resistance to various antibiotics including aminoglycosides, most cephalosporins, aztreonam by virtue of multidrug efflux pumps, and chromosomal OXA-114-like β-lactamases. It also illustrates increased drug resistance to carbapenems by virtue of AmpC type β-lactamases, and metallo-β-lactamases (MBLs) [14]. The MDR nature of bacteria and lack of data on appropriate therapy makes treating these bacteria difficult in hospital settings. Our study intended to recognize the risk factors and underlying comorbidities predisposing to bacteremia by *Achromobacter* spp. and furthermore analyse the pattern of antibiotic resistance among these isolates.

## Methods

### Study design and study population

We performed a retrospective observational study where automated blood cultures positive for *Achromobacter* spp. were assessed for clinical characteristics and antibiotic susceptibility patterns from January 2022 to December 2022 in the microbiology laboratory of a university hospital in Northern India. The study protocol was approved by the Institutional Ethics Committee (2021–52-EMP-EXP Dated 13/04/21). Informed consent was waived off given our study is retrospective.

### Data collection

A set of blood culture bottles were received in the Department of Microbiology and sent to the Bacteriology section for standard microbiological processing like culture using the BACTEC blood culture system (Becton Dickinson Diagnostic Instrument Systems, Sparks, MD) followed by microscopy for bacterial isolation. Matrix-assisted laser desorption/ionization-time of flight mass spectrometry (MALDI-TOF MS) and (VITEK MS, bioMérieux, USA) were the diagnostic modalities used for confirmed identification of *Achromobacter* spp. in this study.

### Inclusion criteria

Only a true pathogen was incorporated in our study. A true pathogen was defined based on the time of positivity of blood culture and clinical and laboratory indexes. Using the National Healthcare Safety Network definitions, the blood cultures that flagged positive when sent to the laboratory within 48 h of admission were labelled as community-acquired BSI and the blood cultures that flagged positive when sent to the laboratory after 48 h of admission were labelled as nosocomial in origin [15].

### Exclusion criteria

Isolates collected only on a single positive culture without any significant clinical parameters were considered a contaminant and excluded from the study.

### Antimicrobial susceptibility testing

Antibiotic susceptibility testing was performed using conventional (Kirby–Bauer disc diffusion method on cation adjusted Müller–Hinton agar) and automated methods (Vitek 2). Due to the non-availability of no standard guidelines for reporting antimicrobial susceptibility testing (AST) for *Achromobacter* spp., we performed AST by using the following antimicrobial agents amikacin (30 mcg), aztreonam (30 mcg), ceftazidime (30 mcg), ceftriaxone (30 mcg), ciprofloxacin (5 mcg), imipenem (10 mcg), meropenem (30 mcg), Piperacillin- tazobactam (100/10 mcg), ticarcillin-clavulanic acid (75/10 mcg), doxycycline (30 mcg) and trimethoprim-sulfamethoxazole (1.25/23.75 mcg).

### Treatment administered

We considered the use of appropriate antibiotics even if one drug from the panel was used in the treatment of the patient.

### Statistical analysis

Categorical variables were described using percentages and a Chi-square test was applied. Univariate analysis of the risk factors in all patients included in our study was assessed using Kaplan–Meier survival analysis. All statistical analyses were performed using SPSS statistical software (IBM SPSS version 20, Armonk, N.Y.).

## Result

A total of 2435 cases of bacteremia were encountered in this study, of which 14 cases (14/2435, 0.57 %) of *Achromobacter* spp. were identified among all bacteria isolated in 1 year. The mean age of the patients included in our study cohort was 37.59±23.17 years with a male predominance (8/14, 57.1 %). We extracted the history of patients from the electronic information system of the institute (Table 1). The most common underlying comorbidities identified in the patients were acute respiratory distress syndrome (ARDS) (7/14, 50.0 %) followed by encephalopathy (7/14, 50.0 %), renal failure (6/14, 42.9 %), hypertension (6/14, 42.9 %) and diabetes mellitus (5/14, 35.7 %). The most common predisposing condition was cytopenia (4/14, 28.57 %) with a subset of neutropenia, which was observed in 3 (3/14, 21.4 %) patients followed by 2 (2/14, 14.28 %) patients each undergoing chemotherapy and hemodialysis.

**Table 1.** Demographic and clinical characteristics of patients with *Achromobacter* bacteremia (*N*=14)

Variables	Values(N,%)
**Age, years (mean±sd**)	37.59±23.17
**Gender (male, *n*%**)	8 (57.1 %)
**Charlson comorbidity score (median, range**)	5(0–7)
**Underlying comorbidities (*n*, %**)	
Hypertension (HTN)	6 (42.9 %)
Acute respiratory distress syndrome (ARDS)	7 (50.0 %)
Diabetes mellitus (DM)	5 (35.7 %)
Renal failure (RF)	6 (42.9 %)
Electrolyte imbalance	5 (35.7 %)
Cerebrovascular disease (CVD)	3 (21.4 %)
Encephalopathy	7 (50.0 %)
Haematological malignancies	2 (14.28 %)
Infective endocarditis	1 (7.1 %)
**Predisposing conditions (*n*, %**)	
Neutropenia	3 (21.4 %)
Undergoing chemotherapy	2 (14.28 %)
Post-operative condition	1 (7.1 %)
Cytopenia	4 (28.57 %)
Alcoholism	2 (14.28 %)
Undergoing hemodialysis	2 (14.28 %)
**Source of bacteremia (*n*, %**)	
Catheter-related	14 (100.0 %)
Intraabdominal infection	0 (0.0 %)
Pneumonia	2 (14.28 %)
Urinary tract infection	0 (0.0 %)
Soft tissue infection (STI)	0 (0.0 %)
**Polymicrobial bacteremia (*n*, %**)	0 (0.0 %)
**Empirical antibiotic therapy (*n*,%**)	
Monotherapy	2 (14.28 %)
Combination therapy	12 (85.72 %)
**Regimen containing**	
Carbapenem	9 (64.28 %)
Third-generation cephalosporin	6 (42.86 %)
Beta-lactam/Beta-lactam inhibitors	6 (42.86 %)
Aminoglycosides	2 (14.28 %)
Vancomycin	2 (14.28 %)
Others	9 (64.28 %)
**30 day all-cause mortality (*n*,%**)	5 (35.71 %)

We also investigated the source of bacteremia in the patients included in our study cohort. Most patients included in the study were suffering from nosocomially acquired bacteremia (10/14, 71.43 %) whereas only a fraction of patients were suffering from community acquired bacteremia (4/14, 28.57 %). All patients (14/14, 100.0 %) in this study were managed on intravenous antibiotics and thus intravenous access in the form of peripheral line catheters, and only 5 (5/14, 35.7 %) patients were managed using central line catheters. Other than catheters, pneumonia due to *Achromobacter* spp. was observed in two patients that were managed on mechanical ventilation (Table 1). No polymicrobial bacteremia or mixed infections were observed in our patient cohort. Empirical antibiotics were also administered after giving samples for blood culture. A total of 12 (12/14, 85.71 %) patients were treated with a combination therapy including meropenem-cilastatin and teicoplanin to cover both the Gram-negative and Gram-positive bacteria. Carbapenems (9/14, 64.28 %) were the most commonly administered antibiotic followed by third-generation cephalosporins (6/14, 42.86 %) and β-lactam/β-lactam inhibitors (6/14, 42.86 %).

**Fig. 1.**
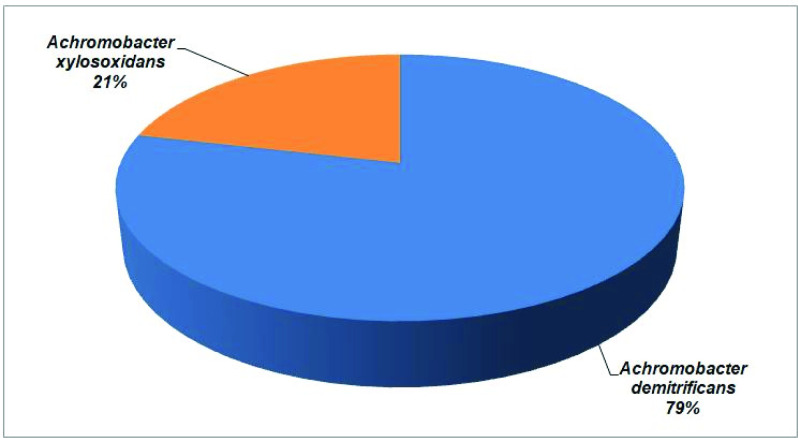
The distribution of Achromobacter denitrificans and Achromobacter xylosoxidans in our study cohort (*n*=14)

We identified all the isolates using MALDI-TOF-MS. The most common species identified in our study was *Achromobacter denitrificans* (11/14, 78.57 %) followed by *Achromobacter xylosoxidans* (3/14, 21.43 %) (Fig. 1). On antibiotic susceptibility testing performed by the Kirby–Bauer disc diffusion method, the most common antibiotic found to be susceptible against *Achromobacter* spp. was ticarcillin-clavulanic acid (14/14, 100.0 %) followed by fluoroquinolones (12/14, 85.72 %) and trimethoprim-sulphamethoxazole (12/14, 85.72 %). *Achromobacter* isolates in this study showed the highest resistance to Azetreonam (12/14, 85.72 %) followed by ceftriaxone (11/14, 78.57 %) and meropenem (8/14, 57.14 %) (Table 2).

**Table 2.** Results of *in vitro* susceptibility testing of *Achromobacter* spp. isolates (*n*=14)

Agents	Susceptible (%)	Intermediate (%)	Resistant (%)
**Amikacin**	7 (50.0 %)	0 (0.0 %)	7 (50.0 %)
**Aztreonam**	2 (14.28 %)	0 (0.0 %)	12 (85.72 %)
**Ceftazidime**	7 (50.0 %)	0 (0.0 %)	7 (50.0 %)
**Ceftriaxone**	3 (21.43 %)	0 (0.0 %)	11 (78.57 %)
**Ciprofloxacin**	12 (85.72 %)	0 (0.0 %)	2 (14.28 %)
**Doxycycline**	9 (64.28 %)	1 (7.14 %)	4 (28.57 %)
**Imipenem**	6 (42.86 %)	3 (21.43 %)	5 (35.71 %)
**Meropenem**	6 (42.86 %)	0 (0.0 %)	8 (57.14 %)
**Piperacillin – tazobactam**	8 (57.14 %)	0 (0.0 %)	6 (42.86 %)
**Ticarcillin – clavulanic acid**	14 (100.0 %)	0 (0.0 %)	0 (0.0 %)
**Trimethoprim - sulphamethoxazole**	12 (85.72 %)	0 (0.0 %)	2 (14.28 %)

The mortality observed in our study was 35.7 % (5/14, 35.7 %). We compared the demographic and clinical characteristics among the *Achromobacter* bacteremia patient who died and survived in Table 3. Among the underlying comorbidities, electrolyte imbalance and cerebrovascular disease in the form of stroke were statistically significant risk factors associated with death in our study cohort. Neutropenia (*P* value=0.009) was identified as the most significant predisposing factor responsible for increased death in *Achromobacter* bacteremia patients. Empirical administration of antibiotics was done in patients after blood culture samples were taken. Carbapenems administration (*P* value=0.038) was more significantly associated with patients that died. Procalcitonin levels (*P* value=0.05) were found to be significantly raised in patients who died.

**Table 3.** Comparison of demographic and clinical characteristics among the *Achromobacter* bacteremia patient who died and survived (*n*=14)

Variables	Alive (*n*=9)	Dead (*n*=5)	p-value
**Age, years (mean±sd)**	40.69±28.72	48.00±13.95	0.606
**Gender (male, *n*%)**	5 (55.56 %)	3 (60.0 %)	0.872
**Charlson comorbidity score (mean, sd)**	4.33±2.18	5.00±1.41	0.550
**Underlying comorbidities (*n*, %)**			
Hypertension (HTN)	4 (44.44 %)	2 (40.0 %)	0.872
Acute respiratory distress syndrome (ARDS)	4 (44.44 %)	3 (60.0 %)	0.577
Diabetes mellitus (DM)	3 (33.33 %)	2 (40.0 %)	0.803
Renal failure (RF)	3 (33.33 %)	3 (60.0 %)	0.334
Electrolyte imbalance	1 (11.11 %)	4 (80.0 %)	0.010*
Cerebrovascular disease (CVD)	0 (0.0 %)	3 (60.0 %)	0.009*
Encephalopathy	6 (66.67 %)	1 (20.0 %)	0.094
Haematological malignancies	1 (11.11 %)	1 (20.0 %)	0.649
Infective endocarditis	0 (0.0 %)	1 (20.0 %)	0.164
**Predisposing conditions (*n*, %)**			
Neutropenia	0 (0.0 %)	3 (60.0 %)	0.009*
Undergoing chemotherapy	1 (11.11 %)	1 (20.0 %)	0.649
Post-operative condition	0 (0.0 %)	1 (20.0 %)	0.164
Cytopenia	3 (33.33 %)	1 (20.0 %)	0.597
Alcoholism	1 (11.11 %)	1 (20.0 %)	0.649
Undergoing hemodialysis	1 (11.11 %)	1 (20.0 %)	0.649
**Source of bacteremia (*n*, %)**			
Central line catheter-related	2 (22.22 %)	3 (60.0 %)	0.158
Intraabdominal infection	0 (0.0 %)	0 (0.0 %)	–
Pneumonia	1 (11.11 %)	1 (20.0 %)	0.649
Urinary tract infection	0 (0.0 %)	0 (0.0 %)	–
Soft tissue infection (STI)	0 (0.0 %)	0 (0.0 %)	–
**Polymicrobial bacteremia (*n*, %)**	0 (0.0 %)	0 (0.0 %)	–
**Empirical antibiotic therapy (*n*,%)**			
Monotherapy	2 (22.22 %)	0 (0.0 %)	0.255
Combination therapy	7 (77.78 %)	5 (100.0 %)	0.255
**Regimen containing**			
Carbapenem	4 (44.44 %)	5 (100.0 %)	0.038*
Third-generation cephalosporin	5 (55.56 %)	1 (20.0 %)	0.198
Beta-lactam/Beta-lactam inhibitors	5 (55.56 %)	1 (20.0 %)	0.198
Aminoglycosides	2 (22.22 %)	0 (0.0 %)	0.255
Vancomycin	1 (11.11 %)	1 (20.0 %)	0.649
Others	6 (66.67 %)	3 (60.0 %)	0.803
**Appropriate antibiotics administered**	6 (66.67 %)	4 (80.0 %)	0.597
**Length of hospital stay (in days)**	15.22±11.22	12.00±5.61	0.564
**Total leukocyte count (mean, sd) in cells/cubic mm**	18790±12 209	12980±9841	0.382
**Procalcitonin levels (mean, sd) in ng ml^−1^**	3.12±9.37	15.65±12.68	0.05*
**Nosocomially acquired infection**	7 (77.78 %)	3 (60.0 %)	0.480
**Community-acquired infection**	2 (22.22 %)	2 (40.0 %)	0.480

*p-value ≤ 0.05 is statistically significant

**Fig. 2.**
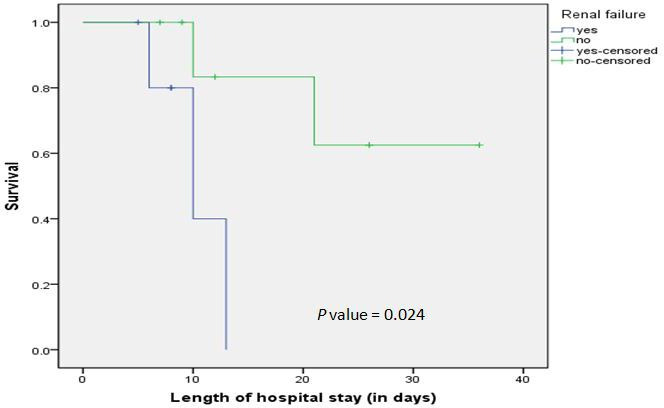
KaplanMeier urvival analysis of 40 day survival of the *Achromobacter* bacteremia patients suffering from renal failure in comparison to patients not suffering from renal failure (*N*=14)

The all-cause 40 day mortality was observed in 35.7 %(5/14, 35.7 %) with two deaths, which were directly attributable to sepsis. We applied the Kaplan–Meier survival analysis on various risk factors observed in *Achromobacter* bacteremia and found renal failure (*P* value=0.024) as a significant risk factor in patients who died due to *Achromobacter* bacteremia (Fig. 2).

## Discussion

The incidence of *Achromobacter* spp. *bacteremia* encountered in this study was 0.57 % (14/2435, 0.57 %), which corroborates with a study by Pandey *et al.* [16], which illustrated an incidence of *Achromobacter* spp. infections as 0.46 % (63/13831, 0.46 %). The mean age of patients was 37.59±23.17 years with a male predominance (8/14, 57.1 %) corroborating with a study by Barragán *et al.* [14]. In this single-centre study, we observed a high incidence of acute respiratory disease (ARDS) (7/14, 50.0 %), Encephalopathy (7/14. 50.0 %), renal failure (6/14, 42.86 %) and hypertension (6/14, 42.86 %) in contrast to a study by Barragán *et al.* [14] where the most predominant comorbidity was underlying malignancy and we observed only 2 (2/14, 14.28 %) cases of haematological malignancies. Catheter-related bacteremia (14/14, 100 %) was predominantly observed as the source of bacteremia, which is in agreement with Barragán *et al.* [14].

*Achromobacter xylosoxidans* is an unusual pathogen in many large studies involving the analysis of Gram-negative bacteria isolated from bloodstream infections [17, 18]. Other species of *Achromobacter* and *Alcaligenes* are known as causative agents in less than 2 % of cases of bacteremia [19, 20]. Three (3/14, 21.43 %) cases of *Achromobacter xylosoxidans* and 11(11/14, 78.57 %) cases of *Achromobacter denitrificans* (Fig. 1) were isolated from bloodstream infections in this study. The cause of rare cases of *Achromobacter* bacteremia could be attributed to the absence of these micro-organisms from the normal skin flora of patients with immunocompromised conditions that could get infected bacteria from their skin flora [21, 22].

Studies by Igra-Siegman *et al.* [23] and Vartivarian *et al.* [22] reported urinary tract infections with *Achromobacter* spp. while we did not encounter any cases of urinary tract infections in our study cohort. Pneumonia caused by this micro-organism was attributable to high case fatality among the patients. We identified 2 (2/14, 14.28 %) cases of pneumonia attributable to secondary bacteremia with *Achromobacter* spp. both were intubated during their hospital stay and further succumbed to their infections as a case fatality rate of 67 % was reported in a study by Morrison *et al.* in patients suffering with *Achromobacter* pneumonia [24].

Intravenous catheters were present in all 14 patients and removal of peripheral intravenous catheters was found to be effective in 7 (7/9, 77.78 %) out of 9 patients on intravenous access from peripheral sites whereas 2 patients succumbed to their ailment. Five (5/14, 35.71 %) patients were managed on central line catheters and did not undergo removal of the catheter, and were managed on antibiotics with an outcome of 60 %(3/5, 60.0 %) mortality. Similarly, bacteremia was observed to recur and high mortality was noted in patients with a catheter *in situ* with only 2 (2/5, 40.0 %) patients that were treated only on antibiotic therapy. Procalcitonin levels (*P* value=0.05) were found to be significantly raised in patients who died, owing to the fact that procalcitonin is denoted as a marker of sepsis, which is recognized as a common cause of death in hospitalized patients [25].

*Achromobacter* spp. is known to be resistant to aminoglycosides, older cephalosporins and narrow-spectrum penicillins and susceptible to sulphonamides, third-generation cephalosporins, and broad-spectrum penicillins and variably susceptible to fluoroquinolones [26, 27]. The *Achromobacter* spp. isolated from the bloodstream of patients included in our study was 100 %(14/14, 100.0 %) susceptible to ticarcillin–clavulanic acid, 85.72 %(12/14, 85.72 %) susceptible to trimethoprim–sulphamethoxazole (cotrimoxazole) and ciprofloxacin. Most *Achromobacter* spp. (8/14, 57.14 %) isolated from blood culture samples in our study showed *in vitro* susceptibility to piperacillin–tazobactam, which corroborates with studies by Turel *et al.* [28] and Nakamoto *et al.* [29]. We also reported a case of prosthetic valve *Achromobacter* endocarditis susceptible to piperacillin–tazobactam and trimethoprim–sulphamethoxazole, which corroborates with the susceptibility pattern of a previous reported case from our institute [30] and a case series by Liu *et al.* [31]. We come across *Achromobacter* spp. isolates as highly (12/14, 85.72 %) susceptible to trimethoprim–sulphamethoxazole, which agrees with studies by D'Amato *et al.* [32] and Olson *et al.* [33]. In contrast to the increasing resistance to fluoroquinolones in recent studies by Isler *et al.*, all (14/14, 100 %) isolates in our study were susceptible to ciprofloxacin [34]. Carbapenems administration (*P* value=0.038) was more significantly associated with patients that died, which could be attributed to various efflux pump mechanisms and metallo-β-lactamases leading to extrusion or inactivation of the drugs rendering it ineffective [35].

The overall mortality observed in our study was 35.71 % (5/14, 335.71 %). We performed Kaplan–Meier survival analysis on the patients with *Achromobacter* bacteremia in our study cohort and identified the absence of renal failure in the patient cohort as a statistically significant cause of increased survival in *Achromobacter* bacteremia patients. A similar outcome with a mortality rate of 33.33 %(5/15, 33.33 %) was observed in a study by Liu *et al.* [31].

The limitations of our study firstly include its retrospective nature, which can lead to the introduction of information bias into the study. Secondly, the data represented in this study only signifies the prevalence of *Achromobacter* bacteremia at a single hospital and does not represent the incidence of *Achromobacter* bacteremia in the geographical region.

## Conclusion

This study provides insight to clinicians into the incidence of *Achromobacter* bacteremia at our centre and the necessary antibiotic therapy to combat it. This isolate is rarely identified from bloodstream infections in immunosuppressed patients and renal failure was identified as a prominent risk factor leading to increased 40 day mortality.
